# Optimization of Fermentation Conditions for Reteplase Expression by *Escherichia coli* Using Response Surface Methodology

**Published:** 2019

**Authors:** Hamze Zare, Hamid Mir Mohammad Sadeghi, Vajihe Akbari

**Affiliations:** Department of Pharmaceutical Biotechnology and Isfahan Pharmaceutical Research Center, Faculty of Pharmacy, Isfahan University of Medical Sciences, Isfahan, Iran

**Keywords:** Bioreactors, *Escherichia coli*, Fermentation, Reteplase

## Abstract

**Background:**

Expression of heterologous proteins at large scale is often a challenging job due to plasmid instability, accumulation of acetate and oxidative damage in bioreactors. Therefore, it is necessary to optimize parameters influencing cell growth and expression of recombinant protein.

**Methods:**

In the present study, the optimal culture conditions for expression of reteplase by *Escherichia coli* (*E. coli)* BL21 (DE3) in a bench-top bioreactor was determined. Response Surface Methodology (RSM) based on Box-Behnken design was used to evaluate the effect of three variables (*i.e*., temperature, shaking speed and pH) and their interactions with cellular growth and protein production. The obtained data were analyzed by Design Expert software.

**Results:**

Based on results of 15 experiments, a response surface quadratic model was developed which was used to explain the relation between production of reteplase and three investigated variables. The high value of “R-Squared” (0.9894) and F-value of 51.99 confirmed the accuracy of this model. According to the developed model, the optimum fermentation conditions for reteplase expression were temperature of 32*°C*, shaking speed of 210 *rpm*, and pH of 8.4. This predicted condition was applied for the production of reteplase in the bioreactor leading to a protein yield of 188 *mg/l*.

**Conclusion:**

Our results indicate the significant role of culture conditions (*e.g*., pH, temperature and oxygen supply) in protein expression at large scale and confirm the need for optimization. The proposed strategy here can also be applied to experimental set-up of optimization for fermentation of other proteins.

## Introduction

Reteplase is a recombinant version of tissue Plasminogen Activator (tPA), a proteolytic enzyme (Serine protease) which converts plasminogen to plasmin and dissolves blood clots (thrombolysis) 1. Reteplase, a mutant of alteplase, contains 357 of the 527 amino acids of tPA and consists of kringle 2 and protease domains of the parent molecule. The kringle 2 domain is responsible for specific binding of reteplase to fibrin rich clots, while the protease domain degrades the fibrin matrix through converting plasminogen to plasmin 2. Reteplase was approved by the Food and Drug Administration (FDA) in 1996 for management of acute myocardial infarction 3. It is a third-generation of thro-mbolytics and in comparison with alteplase has lower affinity to fibrin leading to its better penetration into clots and subsequently lower incidence of bleeding. Furthermore, reteplase has longer half-life compared with alteplase making it more rapid, easy and safe to be administrated 4,5. This molecule lacks carbohydrate side chains of the original molecule, therefore it can be easily expressed by *Escherichia coli (E. coli)* 5. However, other expression systems including *Pichia pastoris (P. pastoris)*, baculo virus-insect cell, tobacco plants were used for production of reteplase 6–8.

*E. coli* is a common host which is widely used for the production of heterologous proteins practically non-glycosylated ones 9. This protein expression platform has some advantages including rapid growth on inexpensive substrates, easy obtaining of high cell densities and well-studied genetic and physiological properties 10. Moreover, *E. coli* can be easily manipulated and has a large number of cloning vectors and various cultivation strategies. Considering these superiorities, *E. coli* can be a suitable host for large-scale manufacturing of heterologous proteins 11.

Large-scale bioprocess of proteins in bioreactor provides well-controlled culture environment [*e.g*., pH value, temperature, shear forces and the Dissolved Oxygen (DO) concentration) 12. It is necessary to optimize fermentation conditions for improvement of the yield and efficiency of protein production in a bioreactor 13. There are different statistical design approaches for optimization of the fermentation process. One of these methods is one-variable-at-a-time optimization where one parameter is varied but others are kept constant. This approach has some disadvantages such as requiring numerous experiments, being laborious and time-consuming and being unable to identify the interactions between the variables which cause misinterpretation of the results 14,15.

Response Surface Methodology (RSM) is an alternative approach being able to describe the possible interactions between experimental variables and accurately predict the optimal condition 16. Although RSM is widely used for agriculture, engineering and food, this method has been also utilized for the optimization of bioprocesses 15–17. The aim of this study was to optimize fermentation conditions (*e.g*., temperature, pH and rotating speed) of reteplase in a bench-top bioreactor using RSM.

## Materials and Methods

### Microorganism and plasmid

BL21 (DE3), *E. coli* str. B F^–^*ompT gal dcmlonhsdS_B_* (*r_B_^−^m_B_^−^*) λ(DE3 [*lacI lacUV5-T7p07 ind1 sam7 nin5*]) [*malB^+^*] _K-12_(λ^S^) (Novagen, USA) was used as the bacterial host for the expression of reteplase. This strain was transformed with the recombinant plasmid (pDset-527-Ret) using heat shock method. pDset-527-Ret was used as an expression vector containing the synthetic gene of reteplase tagged with hexa histidine whose expression was under the control of Lac promoter.

### Small scale expression

A single positive colony was inoculated into Luria-Bertan (LB) broth containing ampicillin (100 *μg/ml*) and incubated at 37*°C* overnight. Then, this culture was used to inoculate (10% *v/v*) 50 *ml* of fresh LB broth and incubated at 37*°C* and 180 *rpm* to reach an optical density (OD_600_) of 0.4–0.6. To induce protein expression, isopropyl β-D-1-thiogalactopyranoside (IPTG) to the final concentrations of 1 *mM* was added and the culture was further incubated at 37*°C* for 2 *hr*.

### Sodium dodecyl sulfate polyacrylamide gel electrophoresis (SDS-PAGE) and western blot analysis

Protein samples were prepared and heated to 95*°C* for 5 *min* to disrupt protein structures. The samples were loaded onto a 12% SDS-polyacrylamide gel and separated by electrophoresis. The gel was subjected to Coomassie staining and destaining.

For western blot analysis, separated proteins were transferred on to a nitrocellulose membrane. Then, the membrane was blocked using 3% skim milk and incubated with Anti-6X His tag (HRP) antibody (Abcam, USA, 1:10,000) at ambient room temperature for 1 *hr*. After washing, the membrane was incubated with 3,3’-diaminobenzidine to visualize blots.

### Expression of protein in bioreactor

A 2-L autoclavable glass stirred fermenter (BioG-Micom, Biotron Inc. Korea) with two six-blade Rushton turbines was used for large scale production of reteplase. Frozen glycerol stock was used to prepare pre-inoculum culture and 5 *ml* of it was added to 150 *ml* of fresh LB medium and incubated overnight at 37*°C* and 180 *rpm*. This overnight culture was added to the fermenter containing 1500 *ml* LB broth supplemented with ampicillin under sterile conditions. After reaching to exponential growth phase (OD600 0.4–0.6), the protein expression was induced by addition of IPTG for 4 *hr*. During the entire process, the pH of medium was automatically controlled by adding sterile HCl or Na-OH solution (0.5 N). Propylene glycol as antifoam was added manually when necessary.

### Experimental design and optimization of cultivation by response surface methodology

The effect of three independent variables (*i.e*., pH, temperature and shaking speed) on expression of reteplase by *E. coli* in the bioreactor was evaluated using RSM. Box-Behnken design was applied and each variable was investigated in three levels (−1 as the minimum value, 1 as the maximum value and 0 as the central point). Table 1 presents the level and code of independent variables. Using this design, a set of 15 experimental runs was developed (Table 2) and the obtained data were evaluated by Design Expert software (Version 8.0.7.1, StatEase Inc., Minneapolis, USA).

**Table 1.** Variables and levels used in the experimental design

**Symbol**	**Variable**	**Coded level**			**Unit**

		**−1**	**0**	**1**		
**A**	Temperature	23	30	37	*°C*
**B**	Shaking speed	200	300	400	*rpm*
**C**	pH	6.5	7.5	8.5	-

**Table 2.** Box–Behnken experimental design of 3 factors and 3 levels

**Run**	**A (*°C*)**	**B (*rpm*)**	**C (pH)**	**Protein expression (*μg/ml*)**	**Biomass production, dry weight (*mg/ml*)**
**1**	30	300	7.5	60.1	1.58
**2**	30	300	7.5	65.3	1.63
**3**	23	300	6.5	31.3	1.46
**4**	30	200	6.5	44.4	1.24
**5**	23	300	8.5	94.5	1.6
**6**	37	200	7.5	74.5	1.32
**7**	30	400	8.5	143.2	1.5
**8**	37	400	7.5	60	1.7
**9**	30	300	7.5	69.2	1.62
**10**	30	400	6.5	91.4	1.8
**11**	37	300	8.5	140.7	1.42
**12**	23	200	7.5	56.2	1.26
**13**	23	400	7.5	43.7	2.04
**14**	30	200	8.5	185.2	1.42
**15**	37	300	6.5	37.2	1.6

### Analytical methods

To determine cell growth and protein expression, samples were taken every 1 *hr* during the bioreactor culture. OD600 of samples was evaluated to measure the growth of culture and biomass production. Protein expression in each sample was monitored using 12% SDS-PAGE. To estimate amount of protein expression, the intensity of corresponding bond was evaluated using Gel Analyzer 2010 software (Lazar Software, Hungary).

At the final stage of protein expression, bacterial cells were harvested by centrifugation at 7,000×*g* for 15 *min*. The cell disruption was performed using sonication and soluble and insoluble fractions were separated by centrifugation. The insoluble fraction (*i.e*., inclusion bodies) was solubilized using denaturing agent and refolded using stepwise dialysis. The sample was purified using Ni-NTA affinity column, as described previously 18. Then, 20 *μl* of the sample was applied for determining biological activity by Assay Sense Human tPA Chromogenic Activity Kit (Assaypro, USA). The commercial reteplase (Retelies®) was used as a positive control and the buffer as a negative control 19.

## Results

### Small scale expression of reteplase

**Figure 1.**
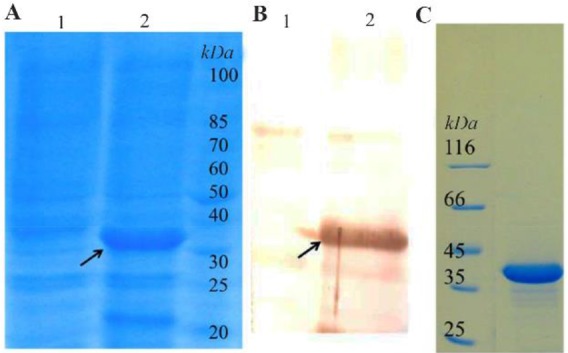
Expression of reteplase induced by 1 *mM* IPTG at 37*°C* in shake flask. A) SDS-PAGE analysis; Lane 1: Uninduced total bacterial protein; Lane 2: Induced total bacterial protein. B) Weston blot analysis; Lane 1: Uninduced total bacterial protein; Lane 2: Induced total bacterial protein. C). SDS-PAGE analysis of commercial reteplase.

Bacterial cell harboring pDset-527-Ret plasmid was used for the expression of reteplase using IPTG induction (1 *mM*) at 37*°C* at shake flask scale. Protein expression was evaluated by SDS–PAGE and western blotting. Figure 1 shows expression of reteplase with an approximately 39 *kDa* molecular weight. Western blotting confirmed that the expressed protein is a Histagged protein (Figure 1).

### Experimental design and modeling for production of reteplase

The experimental design applied in the present work gave 12 runs in triplicates at center points. As a result, a total of 15 experiments were performed (Table 2). The amount of expressed reteplase along with biomass production (based on measurements of OD_600_ and dry cell weight) was determined at the finial fermentation time (Table 2). The concentration of reteplase was considered as the chosen response to evaluate the effect of three variables on production of reteplase. Based on the result of 15 experiments, the relation between production of reteplase and three investigated variables can be explained by the following quadratic equation:

$$\text{Protein}\;\text{expression}\;(\mu g/\mathrm{ml})=64.87+10.84A-2.75B+44.91C-0.50AB+10.07AC-22.25BC-{23.20A}^{2}+{16.93B}^{2}+{34.25C}^{2}$$

### Model analysis and validation

**Figure 2.**
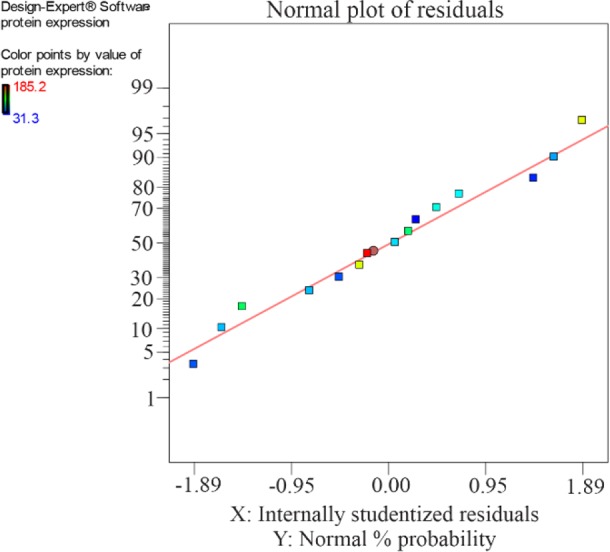
Normal (%) probability plot of the ‘studentized’ residuals for the model of reteplase fermentation.

The statistical significance of the quadratic model was determined by the F-test and the Analysis of Variance (ANOVA) as it is presented in table 3. The high value of “R-Squared” (0.9894) implies goodness of the developed model. The “Pred R-Squared” of 0.8516 is in reasonable agreement with the “Adj R-Squared” of 0.9704, indicating a good correlation according to the model. The “Model F-value” of 51.99 indicates that the model is significant and there is only a 0.02% chance that “Model F-value” this large could occur because of noise.“Adeq Precision” measuring the signal to noise ratio was 24.963. A value greater than 4 indicates that the model can be applied to navigate the design space. Figure 2 presents the normal probability plot indicating a normal distribution of the studentized residuals. Accordingly, this model is a suitable and accurate model for prediction of reteplase expression at different fermentation conditions.

**Table 3.** ANOVA for response surface quadratic model

**Source**	**Sum of squares**	**df**	**Mean square**	**F value**	**Prob> F**	
**Model**	27307.53	9	3034.17	51.99	0.0002	Significant
**A (Temperature)*°C***	939.61	1	939.61	16.10	0.0102	Significant
**B (Shaking speed)**	60.50	1	60.50	1.04	0.3553	Not significant
**C (pH)**	16137.06	1	16137.06	276.49	< 0.0001	Significant
**AB**	1.00	1	1.00	0.0171	0.9010	Not significant
**AC**	406.02	1	406.02	6.96	0.0461	Significant
**BC**	1980.25	1	1980.25	33.93	0.0021	Significant
**A^2^**	1986.63	1	1986.63	34.04	0.0021	Significant
**B^2^**	1058.20	1	1058.20	18.13	0.0080	Significant
**C^2^**	4332.36	1	4332.36	74.23	0.0003	Significant
**Residual**	291.82	5	58.36			
**Lack offit**	250.13	3	83.38		0.2064	Not significant
**Pure error**	41.69	2	20.84			
**Cor total**	27599.35	14				

A, C, AC, BC, A^2^, B^2^ and C^2^ are significant terms of the model (p<0.05). The linear and square terms of temperature, the liner and square terms of pH, the term of interaction between temperature and pH, the term of interaction between shaking speed and pH were significant. The other terms B and AB were not significant in terms of production of reteplase in the fermenter (p> 0.05).

**Figure 3.**
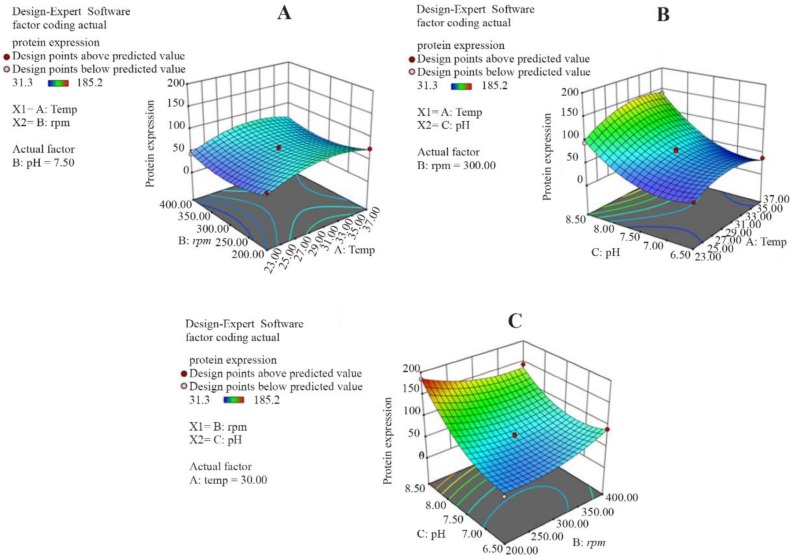
Response surface of reteplase expression represents the interaction between two fermentation variables by keeping other variable constant. A) Interaction between temperature and shaking speed while pH is 7.5; B) Interaction between temperature and pH while shaking speed is 300 *rpm*.C) Interaction between pH and shaking speed while temperature is 30*°C*.

Figure 3 presents the response surface plot of the interaction between two variables while the third one remains constant. As figure 3A shows, production of reteplase increased with an enhancement in temperature, especially when pH is at its high level. As shown in figure 3B, the influence of pH and shaking speed interaction on reteplase production was significant. However, the interaction between temperature and shaking speed (Figure 3C) was not significant (p=0.901).

**Figure 4.**
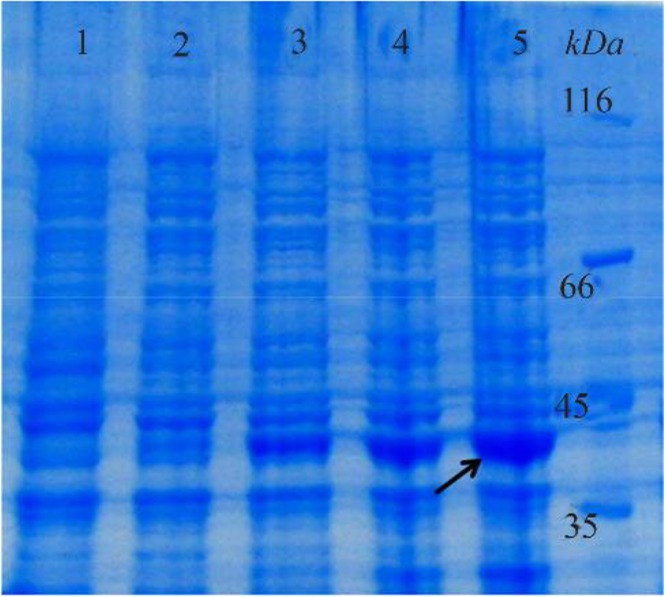
Expression of reteplase under optimum fermentation conditions in a 2-L bioreactor, after 0, 1, 2, 3 and 4 *hr* (Lanes 1–5) of induction with IPTG.

**Figure 5.**
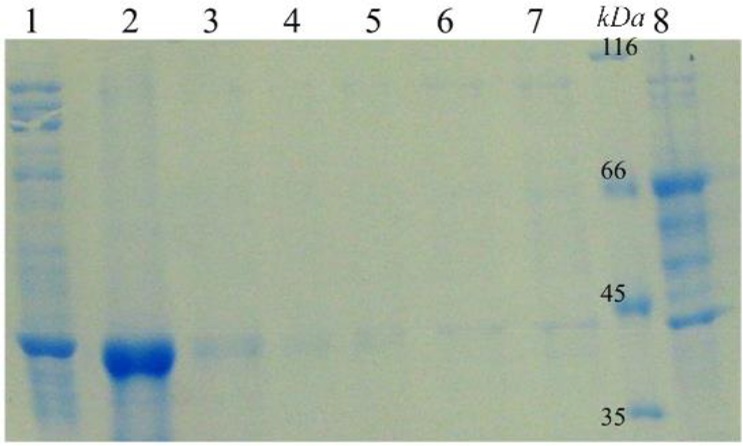
Refolding and purification of reteplase. Lane 1: Refolded protein before adding into column; Lane 2: Eluted protein; Lane 3–7: Wash fractions and Lane 8: Flow-through of column.

According to the developed model, the optimum fermentation conditions for reteplase production were temperature of 32*°C*, shaking speed of 210 *rpm*, and pH of 8.4. This predicted condition was applied for production of reteplase in the bioreactor (Figure 4) leading to a protein concentration of 188 *μg/ml* (with a biological activity of 0.08 *IU*) which was close to the predicted value (186 *μg/ml*). After refolding and purification (Figure 5), the sample was subjected to biological assessment. The specific activity of produced protein was found to be 0.42 *IU* per 1 *mg* of protein which was comparable with the commercial version (0.56 *IU* per 1 *mg* of protein).

## Discussion

The effect of temperature, concentration of inducer and shaking speed on reteplase production in shake flask by *E. coli* BL21 (DE3) was previously evaluated using one-variable-at-a-time optimization 20. However, the scale-up from shake flasks to bioreactors is often a challenging job 21. Plasmid instability, accumulation of acetate and oxidative damage in bioreactors could decrease expression of heterologous protein in large scales 22,23.

Therefore, it is important to optimize parameters which physiologically influence cell growth and expression of recombinant proteins including operating variables (*e.g*., temperature, pH, shaking and aeration), medium components, and supplements (*e.g*., glucose and amino acids) 24. Here, the optimal culture conditions for expression of reteplase by *E. coli* BL21 (DE3) in a bench-top bioreactor were determined using RSM.

RSM is a collection of mathematical and statistical techniques for multivariate optimization of a response (Dependent variable). RSM was used in this study based on Box-Behnken design for optimization of culture conditions. This method can evaluate the effect of different independent variables on a response using a minimum number of experiments. Additionally, in Box-Behnken design, several variables can be simultaneously changed at three levels and the interactions between them can be more efficiently determined 25.

Shaking speed is one of the variables influencing protein expression and cell growth in the bioreactor. According to our results, the most significant expression of reteplase in the fermenter was observed at the lowest shaking speed. These results were in agreement with our previous study in which lower shaking speed resulted in more reteplase expression in shake flask 20. Higher shaking speed can lead to better oxygen supply and therefore higher cell growth. In the present study, lower reteplase expression was observed at higher shaking speeds, while higher agitation rates led to higher cell density. It could be explained based on the fact that a higher cell density can result in more acetate accumulation and less availability of some substances which improve the recombinant protein expression. In agreement with our results, Wang *et al* reported that limitation of oxygen supply resulted in more tPA expression by *E. coli* BL21(DE3) in buffered LB-glycerol medium 26. They suggested lowering oxygen supply and decreasing cell density led to less acetate accumulation and higher ammonium production which was attributed to enhancement of tPA synthesis 26. There are many reports about both increase and decrease of recombinant proteins expression caused by oxygen limitation 27. Therefore, different aeration rates and shaking speeds must be evaluated for each individual protein.

pH is one of the key parameters which can influence expression of membrane and periplasmic proteins and metabolic enzymes 28. Furthermore, pH can affect the activity of bacterial protease and secretory production of proteins 29,30. Here, the effect of pH of growth medium on expression of reteplase was also evaluated which revealed that a pH increase could improve the level of protein production. In agreement with our results, Wang *et al* reported that an alkaline shift can increase expression levels of three examined proteins. They proposed that keeping pH of growth medium around 8 can increase tolerance of *E. coli* BL21(DE3) to acetate stress 26.

Acetate accumulation and acidification of the medium are often observed for prolonged expression of recombinant proteins in LB medium. However, rich cultivation media such as Terrific Broth (TB) show less decrease in pH during growth as they cause higher production of ammonia 31.

Temperature is another critical factor influencing the activity of cellular enzymes, the yield of protein production, the secretion of protein and the solubility of expressed protein 10. In the present study, the optimum temperature for expression of reteplase in the bioreactor was found to be 32*°C*. There are many other reports about better expression of protein at temperature around 30*°C* 32. Practically, the quality of expressed protein at this temperature was higher as the activity and expression of molecular chaperones increased at lower temperature 33–35.

There are few reports about yield of expression of reteplase by *E. coli*. Expression of reteplase in *E. coli* using a helper vector was firstly reported by Kohnert *et al* and overexpression of reteplase as inclusion bodies corresponded to more than 30% of cellular protein 36,37. Another research group reported improvement of soluble expression of reteplase by co-expression of disulfide bond isomerase DsbC yielding 70 *mg* protein per 1 liter of culture 38. Shafiee *et al* described production of reteplase in a stirred tank bioreactor using *E. coli* TOP10 as a host and arabinose as an inducer. They reported the yield of protein expression was 90.5 *mg* per 1 liter of fermentation broth 19. In the present study, 188 *mg* protein was obtained from one liter of bacterial culture. To the best of our knowledge, the results of the current work are the highest protein yield for production of reteplase in *E. coli* in a bench-scale bioreactor.

## Conclusion

In the present work, RSM was applied to evaluate the effect of three fermentation variables including temperature, pH and shaking speed on expression of reteplase by *E. coli* in a bench-top bioreactor. RSM based on Box-Behnken design was utilized to minimize the number of experiments and simplify the data analysis. A wide range of reteplase production (31–185 *μg/ml*) was obtained at 15 excremental runs. These results indicate the significant role of culture conditions (*e.g*., pH, temperature and oxygen supply) in protein expression in large scale and confirm the need for optimization. The highest amount of protein was expressed when fermentation was carried out at temperature of 32*°C*, shaking speed of 210 *rpm* and pH of 8.4. The proposed strategy here can also be applied to experimental set-up of optimization for fermentation of other proteins.
